# Geographical Origin Authentication—A Mandatory Step in the Efficient Involvement of Honey in Medical Treatment

**DOI:** 10.3390/foods13040532

**Published:** 2024-02-09

**Authors:** Tudor Mihai Magdas, Maria David, Ariana Raluca Hategan, Gabriela Adriana Filip, Dana Alina Magdas

**Affiliations:** 1 Department of Anatomy, “Iuliu Hatieganu” University of Medicine and Pharmacy, 3-5 Clinicilor Street, 400006 Cluj-Napoca, Romania; tmmagdas@gmail.com (T.M.M.); adrianafilip33@yahoo.com (G.A.F.); 2 National Institute for Research and Development of Isotopic and Molecular Technologies, 67-103 Donat Street, 400293 Cluj-Napoca, Romania; maria.david@itim-cj.ro (M.D.); ariana.hategan@itim-cj.ro (A.R.H.)

**Keywords:** honey, natural superfoods, geographical origin, botanical origin, rare earth elements, mass spectrometry, clinical trials

## Abstract

Nowadays, in people’s perceptions, the return to roots in all aspects of life is an increasing temptation. This tendency has also been observed in the medical field, despite the availability of high-level medical services with many years of research, expertise, and trials. Equilibrium is found in the combination of the two tendencies through the inclusion of the scientific experience with the advantages and benefits provided by nature. It is well accepted that the nutritional and medicinal properties of honey are closely related to the botanical origin of the plants at the base of honey production. Despite this, people perceive honey as a natural and subsequently a simple product from a chemical point of view. In reality, honey is a very complex matrix containing more than 200 compounds having a high degree of compositional variability as function of its origin. Therefore, when discussing the nutritional and medicinal properties of honey, the importance of the geographical origin and its link to the honey’s composition, due to potential emerging contaminants such as Rare Earth Elements (REEs), should also be considered. This work offers a critical view on the use of honey as a natural superfood, in a direct relationship with its botanical and geographical origin.

## 1. Introduction

**Figure 1:**
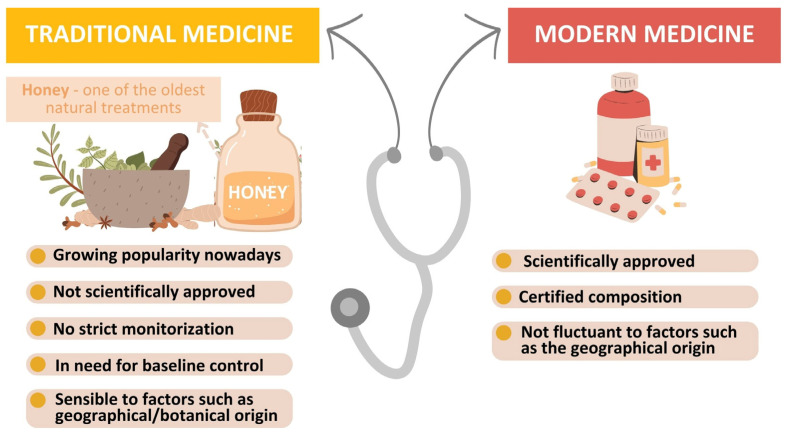
Acknowledged treatment vs. traditional remedies that use honey-based products.

The field of medicine has changed drastically over the past few decades, concurrent with the discovery of new medicines, vaccines, and treatments. Consequently, expectations and quality of life have drastically improved. Despite these advances, there are still some illnesses that are difficult to cure or keep under control (such as diabetes, autoimmune diseases, cancer, and infectious diseases). For this reason, people seek various traditional solutions that can increase the effectiveness of drugs and reduce their toxicity and are often dependent on social, cultural, or religious factors [1,2]. Traditional medicine, as defined by the World Health Organization, is the sum of the skills and practices based on the beliefs and experiences generated from the medical practices of different cultures applied in the maintenance of health as well as in the prevention, improvement, or treatment of physical and mental illness. When adopted outside of its culture, traditional medicine is often referred to as “complementary and alternative medicine” that uses herbs, minerals, bioactive compounds extracted from plants, and other natural products [2,3]. By contrast, modern medicine is an integrated system of practices that deals with the prevention, diagnosis, and treatment of diseases based on scientific knowledge of the human body. Thus, modern medicine mostly uses chemically obtained drugs with stable and balanced compositions that were previously tested in relevant environments and trials and passed rigorous tests according to the in-force legislation [4] (Figure 1).

Nowadays, an increasing number of people from developing and developed countries rely on the association between traditional and modern medicine to meet their primary healthcare needs. The increased acceptance of traditional and alternative medicine arises from the unavailability, high costs, and adverse side effects associated with modern medicine for the treatment of different serious diseases (e.g., diabetes, ulcers, and tuberculosis) or health problems (e.g., injuries, infections, and constipation) [5]. Even though natural remedies have proven their health benefits since antiquity, they are not rigorously regulated, their purity is difficult to standardize, they are based on natural compounds, and they therefore have a high degree of natural variability [6] given by factors such as their botanical and geographical origin. These facts lead to a lack of repeatability and testability compared with modern therapies for products developed based only on natural products (Figure 1) [1,2,4,5,7].

Honey is considered a traditional remedy, and some evidence shows that honey has been widely used for medicinal purposes since the Stone Age [8]. Honey is traditionally used to treat or improve the symptoms of eye diseases, bronchial asthma, throat infections, tuberculosis, hepatitis, ulcers, fatigue, dizziness, constipation, eczema, and wounds [7,9]. Usually, these benefits are directly associated with the botanical origin of honey, the floral source directly related to the honey’s composition, and its medicinal and nutritional benefits. Several studies have reported different antioxidant activities of honey collected from the same area of production as a function of its botanical provenance. Thus, in the study performed by Escuredo et al. [10] on a set of 187 samples of honey having different botanical origins (chestnut, eucalyptus, black berry, heather, honeydew, and polyfloral honeys), honeydew and chestnut honey from the European Atlantic area proved to have the highest mineral, protein, and flavonoid contents as well as the highest antioxidant activity. Sundarban honey possesses protective activity against hydrogen peroxide, UV-irradiation-induced damage, and dose-dependent effects related to DNA protection [11].

Apart from the botanical origin of honey, another factor that strongly contributes to its composition and properties is its geographical origin, especially due to the influence of soil contaminants. This attribute plays a crucial role in the assessment and is directly reflected in the nutritional and medicinal properties of honey. Thus, honey originating from different geographical areas possesses a very distinct physicochemical composition in terms of phenolic, flavonoid, and mineral contents, which can influence honey’s health benefits [12]. In this regard, the antioxidant activity of Polish honey is slightly higher than that of honey originating from Slovakia [13]. Kenyan honey has biochemical content and bioactivities (antioxidant and antimicrobial) comparable to those of intensively studied Manuka honey originating from New Zealand [14]. Therefore, when the nutritional and medicinal benefits of honey are discussed, it is mandatory to refer not only to its botanical source but also geographical origin. This is because the natural compositional differences among types of honey having the same botanical sources, but distinct geographical origins are quite important, and some examples will be presented hereafter [15]. Special reference will be made to the impact of soil elements, especially rare elements, on the beneficial properties of honey.

A very high degree of interest in the development of new approaches for authentication of the geographical origin of honey has been manifested by research groups worldwide [16,17,18], but mainly in the context of food control issues. Despite this, most of these studies were performed in the context of food control areas without any interest in the differences in terms of therapeutic effects. The most effective approach for the assessment of geographical origin is the combination of Isotope Ratio Mass Spectrometry (IRMS) and Inductively Coupled Plasma Mass Spectrometry (ICP-MS), either through advanced statistical methods or artificial intelligence. Isotope ratio determinations of the so-called “bioelements” (H, O, C, N, and S) are widely recognized markers for the assessment of the geographical origin of honey. This is because the isotopic signatures of natural products are directly related to geographical conditions such as climate, geology, and precipitation. In addition, the elemental content determined using ICP-MS reflects the natural soil composition, agricultural practices (fertilizers and pesticides), and pollution from a certain area. Some of these metals, such As, Hg, Cd, and Pb, have been proven to be toxic [19,20]; thus, allowable limits in food were established at the EU level. According to Sarsembayeva et al. [21], 90% of commonly studied food products contain toxic elements that lead to a five-fold increase in the level of heavy metals in raw food materials.

**Figure 2:**
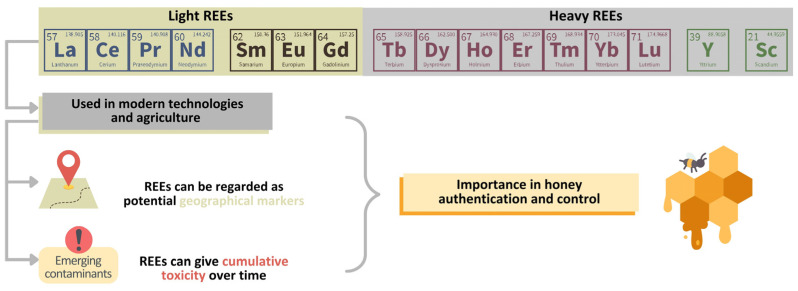
The Rare Earth Element (REE) group (between tracers and contaminants).

In addition, there are other emerging contaminants, such as Rare Earth Elements (REEs), to which special attention should be paid. REEs constitute a group of chemical elements including lanthanides, yttrium, and scandium (Figure 2). These play a critical role as essential raw materials in a spectrum of low-carbon technologies, making them indispensable to the ongoing transition toward sustainable energy and environmental practices. Consequently, in recent years, an increasing number of projects have been dedicated to the exploration of REEs and the establishment of processing plants [22], resulting in a significant increase in REE concentrations in the environment, which is expected to continue in the coming decades [23].

Simultaneously, there has been a growing focus on the toxicological aspects of REEs and, in this regard, recent literature data point to potential correlations between REE exposure and adverse health consequences. These consequences include cardiovascular and central nervous system injuries, infertility, and congenital anomalies [24]. Physiological investigations utilizing animal models have revealed the capacity of REEs to accumulate within the organism, provoke inflammation and oxidative stress, and profoundly disrupt cellular metabolism [25,26]. It has also been shown that REEs are important geographical discriminants as a particularity of a specific growing area [27]. Even if many of these compounds or minerals do not have an unfavorable influence on health, some REEs have been proven to be associated with certain diseases, such as lymphoma in children from China [28]. Based on analytical data generated through IRMS and ICP-MS (stable isotope ratios of the light elements and the elemental profile), either individually or in combination, effective models for origin differentiation have been reported in the literature. Among the markers based on which models for geographical origin differentiation were possible to develop, the REE content, especially the light REEs, proved to be very effective.

The main aim of these studies was related to the authenticity control of honey in terms of its geographical origin, without further correlation with its medicinal properties. Thus, reliable models for origin discrimination have been reported for honey originating from Romania [15], France [15], Greece [29,30], Italy [31,32], China [33,34], and Brazil [35]. However, these studies were not performed with the aim of identifying the compositional differences in a certain type of honey produced in several countries. Important compositional variability existed, considering that the samples could be discriminated according to their botanical origin.

Therefore, based on the reported data from the literature, a comparison of the distinct therapeutic benefits associated with a certain honey type is difficult to perform because of the large dispersion of composition, as previously stated, with regard to not only its botanical origin but also its geographical origin. This is because the geographical and floral origins are interlinked, thus contributing to the final honey fingerprint. Consequently, certain ambiguities persist and are characterized by conflicting data. An analysis of existing publications reveals the use of a multitude of honey types originating from different sources and administered in varying quantities and durations. A notable lack of standardization in honey administration and the absence of a detailed characterization of the honey type are evident in most studies, which primarily identify the floral type and country of origin. This diversity likely contributes to the disparities in research outcomes. Starting with the above-mentioned considerations, this study paid special attention to the influence of geographical origin on honey composition, with a special focus on the REE content. This particular interest in the presence and concentration of REEs in samples is due to the concerns reported by several research groups [15,35,36,37,38,39,40,41] regarding the toxic effects of these elements and because this subject has not previously been treated in any other paper. In addition, this review aims to evaluate the heterogeneity of honey used in the medical field and identify the methodologies employed thus far to determine the origin and type of honey.

## 2. Materials and Methods

For the present study, a comprehensive literature search was conducted until October 2023 using the US National Library of Medicine National Institutes of Health (PubMed) and Web of Science databases. The primary aim was to provide a comprehensive overview of the available evidence related to the characteristics of honey used in human clinical trials and their correlation with botanical and geographical origin. In addition, special attention was paid to the link between honey composition and rare earth element (REE) content because of the recent findings that associate this element group with health concerns. Therefore, for identifying the most relevant reported studies, a variety of keyword combinations, including “Honey”, “Health”, “Medicine”, and “Human”, was employed. Specifically, within PubMed, the search equation strategy involved the combination of “Honey” [All Fields] AND (“Health” [All Fields] OR “Medicine” [All Fields] OR “Humans” [All Fields]). We obtained 8227 articles; then, filtering by English language yielded 7739 articles, filtering by full text yielded 7181 articles, and filtering by “clinical trial” and “randomized controlled trial” yielded 293 results. Furthermore, the search precision was augmented by incorporating Medical Subject Heading (MeSH) terms, yielding 1856 articles. Filtering by English language yielded 1592 articles, filtering by full text yielded 1414 articles, and filtering by “clinical trial” and “randomized controlled trial” yielded 177 results. Using the PubMed Clinical Queries Tool and the term “Honey” and applying the “Therapy” filter to facilitate the screening of additional articles, we obtained 2570 articles. Filtering by English language yielded 2369 articles, filtering by full text yielded 2162 articles, and filtering by “clinical trial” and “randomized controlled trial” yielded 298 results. In parallel, within the Web of Science database, our search equation was as follows: “Honey” AND (“Human” OR “Health” OR “Medicine”), resulting in 262 articles. Filtering by English language led to 252 articles, and excluding review articles led to 196 articles. After conducting searches in both databases using the aforementioned criteria and tools, duplicate entries were automatically eliminated, leading to 550 distinct articles. Subsequent manual screening of articles based on titles, abstracts, and full-text content led to a final selection of 156 articles.

The specific criteria for including articles in this review were as follows: studies involving humans, clinical trials using honey, full-text articles, and studies published in English. The exclusion criteria included studies on species other than humans, in vitro studies, review articles, inaccessible studies, and studies written in languages other than English.

## 3. Results

A total of 156 studies, consisting of 147 clinical trials involving honey as a therapeutic tool in human subjects and nine articles focused on studying the metabolic response of various honey varieties, were analyzed in the present work. The number of published articles consistently increased, indicating an overall upward trend; 40 articles were published before 2010, while 116 studies were published after 2010. This dataset encompasses studies from around the world, reflecting a broad interest in this research topic. Of the studies we analyzed, 68 studies (43.5%) took place in Asia, particularly Iran, which played a significant role, contributing 25 studies (around 16% of the total). Europe had 38 studies (24.3%), while Africa, North America, Oceania, and South America had 20 studies, 15 studies, 14 studies, and 1 study, respectively.

**Figure 3:**
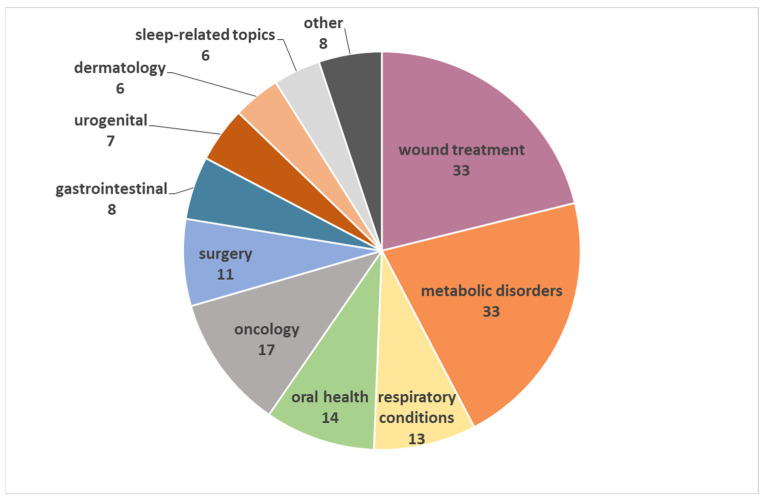
Distribution of medical indications of honey.

The dataset covers a diverse range of medical indications exploring honey as a potential remedy. This distribution is shown in Figure 3.

For this classification, 156 studies were taken into consideration, including wound treatment (33 studies) [42,43,44,45,46,47,48,49,50,51,52,53,54,55,56,57,58,59,60,61,62,63,64,65,66,67,68,69,70,71,72,73,74], metabolic disorders (33 studies) [75,76,77,78,79,80,81,82,83,84,85,86,87,88,89,90,91,92,93,94,95,96,97,98,99,100,101,102,103,104,105,106,107], oncology (17 studies) [108,109,110,111,112,113,114,115,116,117,118,119,120,121,122,123,124], respiratory conditions (13 studies) [125,126,127,128,129,130,131,132,133,134,135,136,137], oral health (14 studies) [138,139,140,141,142,143,144,145,146,147,148,149,150,151], surgery (11 studies) [152,153,154,155,156,157,158,159,160,161,162], gastrointestinal disorders (8 studies) [163,164,165,166,167,168,169,170], urogenital disorders (7 studies) [171,172,173,174,175,176,177], dermatology (6 studies) [178,179,180,181,182,183], sleep-related topics (6 studies) [184,185,186,187,188,189], ophthalmology (5 studies) [190,191,192,193,194], neurocognitive issues (2 studies) [195,196] and hematology (1 study) [197].

Of the 147 clinical trials examined, 124 studies reported a benefit when utilizing honey as an adjuvant therapy, whereas 23 studies did not identify such advantages. Only 18 studies documented the negative or adverse effects associated with the use of honey as a treatment. In 36 studies, it was reported that natural, raw, and local honey were used, while 61 studies used commercial honey either as a food product or incorporated into various medical devices (creams, dressings, and eye gel). However, 59 studies did not mention the honey type used.

Of the analyzed articles, only 81 (51.9%) provided the floral origins of the honeys used. The most frequently employed honey was Manuka honey, found in 24 papers, followed by Tualang and Clover honeys at 11 each. Thyme honey had seven instances, while Acacia, Buckwheat, Kanuka, Citrus, Wildflower, Multifloral, Chestnut, Heather, Pine, and others had lower frequencies, appearing two times or less.

The geographical origin of the honey used was provided in only 39 studies (25%), with 12 studies indicating only the continent or country of origin. Only 25 articles (16%) reported some physical and chemical properties of the materials used, such as pH, viscosity, moisture, density, color, sugar content (glucose, fructose, and fructose–glucose ratio), protein content, phenolic content, water content, diastase, pollen grain count, and metals (copper and zinc), while none reported the possible presence of potentially toxic compounds. Honey was applied locally in 80 cases (51.2%), by oral ingestion in 74 cases (46.8%), and by oral rinsing in 4 cases (2.5%). Three studies used honey through both local application and ingestion. In studies in which honey was used topically, the duration ranged from 1 day to 2 years, with a mean duration of 42 days and a median value of 21 days. For studies using oral rinses for 1–6 weeks, the average duration of application was 17.5 days, with a median of 10.5 days. In studies indicating honey ingestion within a range of 1 day to 12 months, the mean number of days of ingestion was 40.7 days, with a median of 14 days.

The analysis of these 156 articles highlights the diverse nature of clinical studies exploring the therapeutic use of honey. We observed variations in the countries involved, the medical conditions addressed, study outcomes, and the methods and durations of honey utilization. Notably, none of the studies reported the methods used to verify contamination with different elements, and none of the studies utilized tools to verify geographical origin. However, less than a quarter of the studies provided details on the floral and geographical origins of the honey, as well as its physical and chemical properties. This suggests that research in this field is still in its early stages and lacks standardization and uniformity in its approaches.

Based on the previously reported results, it seems that the investigators have not found a significant aspect to thoroughly characterize honey in correlation to its geographical and floral origin when investigating its therapeutic properties, perhaps perceiving honey to be a simpler product. However, honey is a more complex substance, exhibiting significant variations based on its geographical and floral origins, factors that could influence the conclusions of studies investigating the health properties of this natural product. This study underscores the necessity of a comprehensive characterization of the honey used, encompassing floral and geographical origins along with physicochemical attributes.

## 4. Discussion

As more studies have focused on the benefits of honey for human health, there has been both enthusiasm and doubt. Conflicting data can be attributed to the variety of honey types and the lack of reported information, including its properties, origin, and quality. Several studies have suggested that differences in clinical trial outcomes involving honey are attributable to a lack of standardization and the use of diverse honey samples, with important variations in properties and possible active species that are not completely known. However, as the number of clinical trials on honey continues to increase and maintains a consistent upward trend, this issue is expected to be addressed. The increasing number of studies is driven by the accumulation of favorable data with the innovation of using honey, which is characterized by its widespread availability and benefits across multiple indications, and the high level of patient acceptance of natural products.

However, an important disadvantage of natural products, when intended for use as medical treatments, is their variability. Different types of honey have been reported to exhibit slightly different metabolic reactions. To properly assess the health benefits of these products, a full description of the honey used is mandatory. The main reported benefits of honey are related to its antibacterial, antioxidant, and anti-inflammatory properties; but, as we discuss below, these properties are directly related to authenticity issues with the commercial honey, the origin (both botanical and geographical), and the processing steps.

Honey has been incorporated into the recommendations of the World Health Organization (WHO) for pediatric cough management, while specific honey types (e.g., Manuka honey) have received approval from regulatory bodies, such as the Food and Drug Administration (FDA), for their efficacy in wound treatment. The anti-allergic effects of stingless bee honey have proven to be effective as complementary and alternative medicines for the treatment of various allergic diseases [198]. It was reported that Acacia honey has a wound-healing property [199], presented an antiproliferative effect on melanoma human and murine cells [200], and demonstrated anti-tumor activity in lung cancer cells [201]. Manuka honey contains metylglyoxal, a compound that was proven to be responsible for the antibacterial activity of honey [202]. This type of honey also shows synergistic in vitro effects when administered alongside antiviral drugs [203]. In addition, Manuka and Tualang honey presented in vivo effects on breast cancer (a reduction in tumor growth, tumor grading, estrogenic activity, and hematological parameters) [204]. Heather honey demonstrated a greater cytotoxic effect against a human leukemia cell line (HL-60) than rosemary or polyfloral honey [205].

Table 1 summarizes some relevant studies in which the medicinal properties of honey have been scientifically tested.

Several factors influence the antibacterial properties of honey. Bucekova et al. [206] found that natural honey provided by local beekeepers provides a greater antibacterial effect than commercial or medical-grade honeys, while more than 40% of the studied commercial honeys proved to have an antibacterial activity similar to that of artificial products, missing therefore important bee-derived components that grant honey the healing properties. Another study suggested that gamma irradiation of honey, in order to ensure the sterility of the product, decreases its antibacterial properties [69].

Growth suppression of resistant bacterial strains exposed to the same type of honey (Tamarix gallica honey) differed depending on the origin of the honey [207].

It has been reported that the use of medicinal plants, especially honey, is in great demand for cancer patients, despite the medical advice provided by healthcare professionals, owing to the possible interactions between natural products and conventional treatments. Several studies have reported distinct effects of different types of honey on cancer-related complications. For instance, Clover, Thyme, and Astragalus honey treatments were effective in reducing the severity of mucositis in oncological patients, whereas Manuka honey did not produce significant changes in mucositis in similar patients and was less tolerated.

As honey is a natural food and remedy, in addition to the botanical origin, its properties also reflect the environmental conditions, like soil composition, but also pollution and agricultural practices. Despite this, this issue is poorly addressed in the literature; therefore, this review emphasizes the importance of the geographical origin of honey with respect to honey composition (Table 2), from the perspective of an emerging group of pollutants, namely Rare Earth Elements (REEs). These elements are intensively used in technological advances and they are released into the environment through different pathways. Extracted and incorporated into modern technology, these elements expose humans to novel chemical compounds with unknown health effects. Recent studies have reported toxic effects in cellular and animal models, indicating adverse effects on human health. It has been suggested that prolonged exposure to increased concentrations of REEs is associated with an increased risk of leukemia, cardiovascular and central nervous system injuries, infertility, and congenital anomalies in children.

Many studies have reported a high degree of variability in the REE content of honey samples collected worldwide. Thus, the simultaneous determination of the presence of 16 REEs (lanthanum (La), cerium (Ce), praseodymium (Pr), neodymium (Nd), samarium (Sm), gadolinium (Gd), europium (Eu), terbium (Tb), dysprosium (Dy), thulium (Tm), ytterbium (Yb), holmium (Ho), erbium (Er), lutetium (Lu), yttrium (Y), and scandium (Sc)) in the content of honey samples revealed similar values for honey from The Balkans, Kazakhstan, Italy, and South America, while higher values were observed in the composition of Tanzanian honey [37,38]. The lowest Ce content was reported in honey originating from France, Brazil, and Italy, whereas higher values were found in Romanian and Jordanian honey [15,35,36,39]. Regarding the La concentration in honey samples, the lowest values were observed in those produced in Brazil, Greece, and Poland, slightly higher values were determined in Italian honey, and the highest concentration was found in Jordanian honey [29,35,36,39]. These results highlight the importance of considering the type of honey used in specific clinical conditions.

Future research efforts should also integrate extra measures to assess contamination related to novel substances, such as Rare Earth Elements (REEs).

## 5. Conclusions

Studies exploring the health effects of honey have demonstrated a degree of heterogeneity. This variability can be attributed to multiple factors, such as variations in the clinical study design, sample size, and duration of follow-up. However, the utilization of various types of honey is an additional factor contributing to this disparity. The distinct compositions and properties of different honey varieties, rooted in their geographical and botanical origins, are likely to exert unique biological effects. Therefore, it is evident that the type of honey (botanical and geographical) employed in these studies plays a significant role in the observed health outcomes, thereby contributing to the mixed nature of the findings. By addressing these factors in the study of honey, a comprehensive comparison of the results is possible, enabling a better understanding of the valuable nature of honey as a therapeutic tool.

In the future, honey may become more extensively incorporated into modern medical practices, which will necessitate rigorous clinical trials similar to those conducted for pharmaceutical products in order to establish which type of honey provides the greatest benefit in specific clinical situations, the adequate dosage, and the interactions with the standard treatment.

## Figures and Tables

**Table 1:** Therapeutic effects of honey observed in the literature.

No	Honey	Pathology	Results	Ref.
1	Manuka, Tualang	Atopic dermatitis	Faster healing of the skin lesion	[179,182]
2	Honey not specified	Pediatric Gastroenteritis	Reduced diarrheic stools and length of hospital stay	[183]
3	Manuka	Pediatric β-thalassemia major	Reduction of oxidative stress; Improved Hemoglobin; Reduced frequency of necessary blood transfusions	[197]
4	Buckwheat, Tualang, Clover, Multifloral	Metabolic activity	Reduces inflammation, antioxidant activity, improved metabolic and cardiovascular parameters	[83,85,86,88]
5	Tualang	Cognitive function	Improves immediate memory in healthy individuals and learning performance in schizophrenia patients	[195,196]
6	Clover, Thyme, Manuka	Oncology	Improves chemo-radiotherapy-induced mucositis; Relieves pain; Improves hematologic parameters	[110,112,119,123]
7	Manuka	Ophthalmology	Improves dry eye symptoms; Accelerates healing in blepharitis	[190,191,192,193]
8	Apis mellifera, Manuka	Oral Health	Reduces bacterial plaque; Anti-inflammatory and soothing effect after dental procedures	[142,147,148]
9	Acacia, Manuka, Thyme	Upper respiratory tract diseases	Improves rhinosinusitis symptoms, ameliorates acute cough in children, reduces nasal bacterial colonization	[127,128,129,137]
10	Buckwheat, Eucalyptus, Labiatae, Citrus	Sleep Quality	Improves sleep quality in coronary patients and children	[185,186,187]
11	Tualang, Silk-cotton, Thyme, Multifloral	Surgery-related symptoms	Reduces post-operative pain, accelerates wound healing	[154,156,158,159,161]
12	Astragalus	Female urogenital pathology	Alleviates dysmenorrhea pain, reduces inflammation	[176]
13	Beri, Wildflower, Manuka, Multifloral, Tualang	Wound care	Improved pain score, faster healing of surgical wounds, pressure ulcers, diabetic foot ulcers	[47,48,58,60,61,69,70]

**Table 2:** Honey authentication with respect to its botanical or geographical origin, by differentiating among the elemental profiles or isotopic ratios, determined using mass spectrometry analytical techniques.

No	Authentication	Analytical Method	Aim	Ref.
1	botanical/geographical	ICP-MS and IRMS	differentiation among 7 varietal honey types originating in both Romania and France by means of elemental and isotopic profiling and emerging REEs	[15]
2	geographical	ICP-MS	differentiating the honeydew honey produced in three regions in Brazil by 39 elements (including REEs)	[35]
3	botanical/geographical	ICP-MS	discriminating between 18 monofloral and multifloral imported honey samples and 12 multifloral Jordanian local samples (elemental profiling including REEs)	[36]
4	botanical/soil distribution	ICP-MS and ICP-OES	determining the traceability of the honey chain with regard to the honey, flowers, and soil origin by comparing the lanthanide distributions of 17 different monofloral honeys	[39]
5	geographical	ICP-MS	analyzing 40 metals (including REEs) in multifloral honey samples from Tanzania, Italia, Kazakhstan, the Balkans, and South America	[38]
6	botanical	ICP-MS	evaluating the trace elements and REE content in four monofloral honeys	[37]
7	botanical/geographical	ICP-MS	elemental metabolomic determination for honey differentiation according to the geographical (Greek vs Poland) and botanic origin	[29]
8	botanical/geographical/entomological origin of bees	TQ-ICP-MS	determination of the best sample digestion procedure for elemental analysis of Brazilian honeys from two species. Authentication based on elemental and composition profile.	[208]
9	geographical	ICP-MS, IRMS and TIMS	fingerprint determination to link soil, water, and honey composition in order to assess the geographical origin of Argentinean honey	[209]
10	geographical	ICP-MS and INAA	demonstrating that elemental analysis may be useful in differentiating honey sourced from Montana against honey from North and South Dakota	[210]
11	botanical	ICP-MS	measuring the concentration of trace and toxic elements in four unifloral honeys produced in Sardinia	[31]
12	botanical	ICP-MS	classifying Chinese honeys according to their botanical origin by the mineral element content and chemometric methods	[211]
13	geographical	ICP-MS	utilizing the elemental fingerprinting of honey to differentiate New Zealand (NZ) honey from honey of international origin (34 other countries)	[212]
14	botanical	ICP-MS	differentiating the mineral content of 55 honey samples originating from three types (honeydew, buckwheat, and rape honey) from different areas in Poland	[213]
15	botanical	ICP-OES and IRMS	investigating the impact of botanical origin on the stable isotopes and elemental content of Greek unifloral honeys	[30]
16	botanical/geographical	MP-AES	differentiation of the geological origin of honey samples from Central Hungary and Western Transdanubia as well as differentiation among five honey types	[214]
17	botanical/geographical	ICP-MS and IRMS	verifying the relationship between stable isotope ratios together with the elemental content and the geographical and botanical origin of honey	[31]
18	botanical	ICP-MS and IRMS	determination of isotope ratios and element chemical fingerprints in order to distinguish between six Chinese honeys	[215]

## Data Availability

Data is contained within the article.
